# Breeding *Jatropha curcas* by genomic selection: A pilot assessment of the accuracy of predictive models

**DOI:** 10.1371/journal.pone.0173368

**Published:** 2017-03-15

**Authors:** Leonardo de Azevedo Peixoto, Bruno Galvêas Laviola, Alexandre Alonso Alves, Tatiana Barbosa Rosado, Leonardo Lopes Bhering

**Affiliations:** 1 Biology Department, Universidade Federal de Viçosa, Viçosa, Minas Gerais, Brazil; 2 Empresa Brasileira de Pesquisa Agropecuária, Embrapa Agroenergia, Parque Estação Biológica–PqEB s/n, Asa Norte, Brasília, Brazil; Pennsylvania State University, UNITED STATES

## Abstract

Genomic wide selection is a promising approach for improving the selection accuracy in plant breeding, particularly in species with long life cycles, such as Jatropha. Therefore, the objectives of this study were to estimate the genetic parameters for grain yield (GY) and the weight of 100 seeds (W100S) using restricted maximum likelihood (REML); to compare the performance of GWS methods to predict GY and W100S; and to estimate how many markers are needed to train the GWS model to obtain the maximum accuracy. Eight GWS models were compared in terms of predictive ability. The impact that the marker density had on the predictive ability was investigated using a varying number of markers, from 2 to 1,248. Because the genetic variance between evaluated genotypes was significant, it was possible to obtain selection gain. All of the GWS methods tested in this study can be used to predict GY and W100S in Jatropha. A training model fitted using 1,000 and 800 markers is sufficient to capture the maximum genetic variance and, consequently, maximum prediction ability of GY and W100S, respectively. This study demonstrated the applicability of genome-wide prediction to identify useful genetic sources of GY and W100S for Jatropha breeding. Further research is needed to confirm the applicability of the proposed approach to other complex traits.

## Introduction

Currently, many countries have invested a lot of money into researching promising species for biofuel production [1] due to the worldwide concern over the emission of toxic gases, which enhances the greenhouse effect and contributes to climate change [2]. Thus, Jatropha (*Jatropha curcas* L.) has become a potential crop for producing biofuel due to the high oil content found in its seeds and the ability to transform this oil into biofuel [3, 4]. Jatropha has an average of 35% seed oil content, and the oil extracted from the seeds has 24.6% crude protein and 47.2% crude fat [5].

Moreover, Jatropha has several agronomic morphological traits that make it a useful crop for producing biofuel and feeding animals, such as drought tolerance [6], rapid growth, easy propagation [7], the fact that it can be grown at almost all altitudes, and because the plants can produce for more than 50 years [8]. In addition, Jatropha oil has good oxidation stability, low viscosity, and a low pour point, making its oil better than soybean oil and palm oil [9].

Although marker-assisted selection (MAS) has played an important role in plant breeding for disease and pest resistance [10, 11], its application in the improvement of quantitative traits such as grain yield (GY) and seed oil content is challenging because those traits are controlled by numerous loci with small effects. Further, the environment has a large effect on these traits, resulting in small to moderate heritability. Peixoto et al. [12] evaluated 179 half-sib families in Jatropha breeding and revealed low heritability to GY (0.35) and oil content (0.24) by REML analysis.

The complexity of traits related to GY and lack of a simple MAS approach warrant the testing of other breeding approaches. Genomic wide selection (GWS), as proposed by Meuwissen et al. [13], has become an important tool to help breeders in plant and animal breeding due to its performance as a prediction model by associating marker information with phenotypic information [13]. To be applied, GWS should have two types of population: in the training population, individual plants should be genotyped and phenotyped, and in the validation population, individual plants should just be genotyped. The primary difference between GWS and traditional forms of MAS is that in GWS, instead of using QTL mapping and a test of significant markers, all markers are included in both the training and validation populations of the GWS model and that all markers are modeled as random (i.e., not chosen for inclusion in the model based on statistical analysis). By utilizing genome-wide molecular markers, GWS is becoming a promising method for the selection of complex traits in plant breeding programs [14] and has been applied to multiple crops, including wheat, maize, and barley [15–17]. The prediction accuracies of GWS have been reported to be 28% greater than MAS and 95% as accurate as phenotypic selection for a single trait in wheat [18].

A few studies have evaluated the use of GWS in forestry breeding. Wong and Bernardo [19] first evaluated the efficiency of GWS for oil palm breeding using simulated data and demonstrated the importance of improving gain per unit time. Grattapaglia and Resende [20], using deterministic models, analyzed the use of GWS in tree genetic improvement and showed that it has great potential to accelerate breeding. This was confirmed by a simulation study of *Cryptomeria japonica* breeding [21]. The prospects of and challenges for fruit quality and disease resistance have been analyzed in apple breeding strategies using GWS [22]. A first experimental study with *Pinus taeda* demonstrated the value of GWS when the models were used at the relevant selection age in accordance with the breeding zone where marker effects were estimated [23]. Similarly, a first experimental result in eucalyptus showed that GWS is of value in understanding the quantitative trait variation in forest trees and is a powerful tool for applied tree improvement [24]. Although few studies have shown the applicability of GWS in forestry breeding, no reports can be found for Jatropha. Therefore, the objectives of this study were thus to a) estimate the genetic parameters for GY and weight of 100 seeds (W100S) using restricted maximum likelihood (REML); b) compare the performance of GWS methods to predict GY and W100S; and c) estimate how many markers are needed to train the GWS model to obtain the maximum accuracy.

## Material and methods

### Experiment design

#### Germplasm bank experiment

179 Jatropha half-sib families from the Embrapa Cerrados germplasm bank were evaluated in this experiment. The Brazilian region where each family were collected can be found in the S1 Table. It was laid out in the experimental field of Embrapa Cerrados, Planaltina, Distrito Federal, Brazil (15°35’30”S and 47°42’30”W; 1007 m asl). The experiment was implemented in November 2008 in a complete randomized block design with 2 replications and 5 plants per replication. Plants were arranged in rows, with 4 m between rows and 2 m between plants. The half-sib families were evaluated in 5 crop years from 2010 to 2014 for weight of 100 seeds (W100S) and grain yield (GY) [25]. Although the experiment was evaluated for 5 years, only the 2013 evaluation was used to perform the analysis because the diallel experiment was only evaluated in that year. All management practices were based on Dias et al. [26], and they were adapted according to recent research advances regarding Jatropha in Brazil [27, 28].

#### Diallel experiment

The experiment was implemented in November 2011 in a complete randomized block design with 5 replications and 3 plants per plot, with 4 m between rows and 2 m between plants. The diallel experiment was carried out using 3 segregating families, with 14 individuals per family (S2 Table). Segregating families were part of a complete diallel and were formed by crossing the contrasting genotypes of the germplasm bank, which contained genotypes of the following characteristics: nontoxic and susceptible to *Oidium* spp., toxic and resistant to *Oidium* spp., and toxic and susceptible to *Oidium* spp. (S2 Table). Trials were located at the experimental area of Embrapa Cerrados, in Planaltina-DF, Brazil (15°35’30”S and 47°42’30”W, 1007 m asl). Crop management practices, e.g., nutrition and pest and disease control, were carried out to maintain the germplasm bank, as recommended for the species [26, 29]. The experiment was evaluated in 2013 for GY and W100S.

### Genotypic data

Because genotyping is expensive and no chip has been established for Jatropha, only 78 plants were genotyped. Thirty-six plants (the first plant in block one) were from the germplasm bank experiment, and 42 plants (14 plants per crossing) were from the diallel experiment (S3 Table).

Total genomic DNA was extracted from younger leaves using the protocol of [30] with minor modifications. Briefly, 5 g of leaves was ground to a powder in liquid nitrogen, 20 μl of extraction buffer (2% CTAB, 20 mM EDTA, 2% PVP, 1.4 M NaCl, 100 mMTris–HClpH 8.0 and 1% β-mercaptoethanol) was added, and the homogenized samples were incubated at 65°C for 1 h. The supernatant was extracted twice with chloroform:isoamyl alcohol (24:1, v/v) and treated with RNase A (100 mg/ml) at 37°C for 30 min. DNA was precipitated with isopropanol and washed twice with 70% ethanol. Pelleted DNA was air dried, resuspended in 100 μl of sterile ultra-pure water, and stored at -20°C. DNA concentration was measured using a NanoDrop spectrophotometer (NanoDrop Products, Wilmington, DE, USA), and the concentration of each sample was adjusted to 2–5 ng.μ^-1^.

Diversity Arrays techonology (DArT PL) was the company responsible to obtain DarTs and SNPs. Many methods have been developed to reduce genome complexity, however the DArT methods provide a significant advantage via an intelligent selection of genome fraction corresponding predominantly to active genes. This selection is achieved through the use of a combination of Restriction Enzymes which separate low copy sequences (most informative for marker discovery and typing) from the repetitive fraction of the genome. While the initial DArT implementation on the microarray platform involves fluorescent labeling of representations and hybridization to dedicated DArT arrays, the DArTseq method deploys sequencing of the representations on the Next Generation Sequencing (NGS) platforms. The advantage of DArTseq over the array version of DArT is currently limited to applications requiring very high marker densities (tens of thousands of markers). This technology is therefore positioned in the area of high resolution mapping and detailed genetic dissection of traits. As modern breeding, moves rapidly in this direction, especially in larger organizations, DArTseq is increasingly used in crop improvement applications. DArTseq for a new organism starts with optimization of complexity reduction method(s). While the choice of restriction enzyme combinations is large, DArT PL has invested considerable effort in testing various combinations on a significant number of organisms and has developed sets of complexity reduction methods (representations) that are performing quite well compared to other methods. The optimization process usually selects one dominant method of complexity reduction for the crop, but in many cases several methods were identified which offer application-specific advantages. The difference between the methods can be both quantitative (different number of unique fragments in the representation) as well as compositional (different sets of fragments captured in the representations). These differences in representation size and composition translate to different efficiencies in marker detection rate and quality (call rate and reproducibility) and can be further optimized for performance in different applications. It used 1,248 SNPs and DArTs markers.

### Statistical analysis

Genetic and environmental parameters were estimated by restricted maximum likelihood (REML) analysis using the Selegen software [31]. Experiment was evaluated using the follow model:

$$Y=X_{r}+Z_{a}+W_{p}+e$$

where Y is the phenotypic values vector; r is the block effect vector (fixed effect); a is the additive effect vector (random effect); p is the interaction between the block effect and the genotype effect (random effect); e is the residual effect vector (random effect); and X, Z and W are the incidence matrix to the block effect, the additive effect, and the interaction between the block effect and the genotype effect, respectively.

Because the second experiment was a diallel, the dominance effect should be fitted into the mixed model. Therefore, experiment 2 was analyzed using the follow model:

$$Y=X_{r}+Z_{a}+W_{p}+T_{f}+e$$

where Y is the phenotypic values vector; r is the block effect vector (fixed effect); a is the additive effect vector (random effect); p is the interaction between the block effect and the genotype effect (random effect); f is the dominance effect vector (random effect); e is the residual effect vector (random effect); and X, Z, W and T are the incidence matrix to the block effect, the additive effect, the interaction between the block effect and the additive effect, and the dominance effect, respectively.

Genetic diversity was estimated by multidimensional scaling analysis (MDS) [32] using the MASS package in the R software [33]. The distance matrix was estimated based on markers which were identical by state (IBS), and a two-dimensional graphic was plotted based on the distance matrix using the scatterplot3d package in R.

Narrow sense heritability was calculated for each experiment as the additive genetic variance divided by the total phenotypic variance. The genetic variance was calculated using the equation proposed by Falconer et al. [34].

Prediction ability was assessed as the Pearson Correlation of the genomic estimate breeding value (GEBV) and phenotypic value in the validation population.

### Genomic prediction models

Eight GWS methods were used for analysis in the field experiment: RR-BLUP, G-BLUP, Bayesian Ridge Regression (BRR), Bayes A, Bayes B, Bayes Cπ, Bayesian LASSO (BLASSO) and Reproducing Kernel Hilbert Spaces Regression (RKHS). In all models, the phenotypic records were described as

$$y_{i}=\mu +g_{i}+\varepsilon_{i}$$

where $y_{i}=n_{i}^{-1}\sum_{i=1}^{k}y_{ik}$ is the average performance of the i_th_ line; n_i_ is the number of replicates used for computing the mean value of the i_th_ genotype; μ is an intercept; g_i_ is the genetic value of the i_th_ genotype; and ε_i_ is a model residual. The genomic selection models differed in how molecular marker information was included in g_i_.

Three methods used in this work were described by Meuwissen et al. [35]: RR-BLUP, Bayes A and Bayes B. RR-BLUP assumes that each marker had variance equal to V_G_/M, where V_G_ is the genetic variance and M is the number of markers. In the Bayes A method, each effect i is drawn from a normal distribution with its own variance: N(0, $\sigma_{gi}^{2}$); the variance parameters are in turn sampled from a scaled inverted chi-squared distribution. In the Bayes B approach, the prior for the proportion of markers associated with zero phenotypic variance, π, was assumed to be unknown. The other prior hyperparameters for marker variance components in Bayes A and Bayes B were as given by Meuwissen et al. [35].

G-BLUP assumes an equal variance for each marker and uses a genomic relationships matrix among all individuals in a reference set and a test set that allows it to compute the variance components and best linear unbiased predictions (BLUP) from a mixed model [36]. This was achieved by replacing the pedigree-based relationship matrix with the genomic relationship matrix (G) estimated from SNP marker genotypes to define the covariance among breeding values.

BRR assumes that each marker had a variance equal to V_G_/M, where V_G_ is the genetic variance and M is the number of markers. The variance was assigned an inverse chi-square (*σ*^2^∼*χ*^−2^(*S*,*v*)).

Bayes Cπ assumes common marker variances and allows some markers to have no effect [37]. Additionally, Bayes Cπ jointly estimates π from the training data to avoid an incorrect π that can negatively affect prediction accuracy [38].

In the BLASSO method, marker effects are assigned independent Gaussian priors with marker-specific variances ($\sigma_{e}^{2}\tau_{j}^{2}$). At the next level of the hierarchical model, the $\tau_{j}^{2}$s are assigned iid exponential priors $EXP\;[\tau_{j}^{2}|\lambda^{2}]$. At a deeper level of the hierarchy, *λ*^2^ is assigned a Gamma prior with a rate (δ) and shape (r), which, in this study, were the default in the BGLR package in R. Finally, independent scaled inverse chi-square priors were assigned to the variance parameters, and the scale and degree of freedom parameters were set to S_u_ = S_e_ = 1 and d:f_:e_ = d:f_:u_ = 4, respectively. BLASSO is described by De Los Campos et al. [39].

In RKHS, genetic values were viewed as a Gaussian process. When markers and a pedigree were available, genetic values were modeled as the sum of two components:

$$g_{i}=u_{i}+f_{i}$$

where u_i_ is the mean and f_i_ is a Gaussian process with a (co)variance function proportional to the evaluations of a reproducing kernel, K(x_i_, x_j_), evaluated in marker genotypes; here, x_i_ and x_j_ are vectors of marker genotype codes for the i_th_ and j_th_ individuals, respectively. All hyper parameters were assumed following De Los Campos et al. [40].

### Marker density

The effect of numbers of markers on prediction ability was determined through five-fold cross-validation by excluding, after each interaction, the marker that had the smallest effect. Therefore, the number of markers decreased from 1248 to 2. Each interaction was repeated 50 times to avoid sampling bias for markers, and the average of these replications was used to represent the prediction ability of each interaction.

The prediction ability average with standard error as the error bars was plotted versus the number of markers in each interaction using Boxplot. G-BLUP was used to perform these analyses because it was the fastest GWS method.

### Software and computer information

All statistical modeling was performed in R. RR-BLUP and G-BLUP were performed using the rrBLUP package (function mixed.solve and kin.BLUP, respectively). The Bayes A, Bayes B, Bayes Cπ and RKHS models were performed using the BGLR package (function BGLR), and BLASSO and BRR were performed using the BLR package (function BLR).

A total of 20,000 burn-ins (number of iterations before the Bayesian analysis convergence) and 40,000 saved iterations, as obtained from the Markov chain Monte Carlo (MCMC) method, was used in all Bayesian methods. The convergence of Bayesian models was checked by inspecting trace plots of the variance parameters.

Two high-performance computers (12^th^ generation, Intel Xeon E5-26 processor, 3.30 GHz, 64 or 96 GB RAM, 1024 GB hard drive) were used to perform all analyses.

## Results

### Phenotypic analysis

Genetic variance was similar in both experiments for grain yield (GY), and 3 times greater in experiment 1 for weight of 100 seeds (W100S) (Table 1). The heritability was moderate for GY in experiment 1 and overestimated in experiment 2. Conversely, the heritability for W100S was overestimated in experiment 1 and moderate in experiment 2. CV_e_ was high and low for GY and W100S, respectively. CV_r_ was greater than 1 for W100S in experiment 1, but it was lower than 1 for GY in the same experiment.

**Table 1:** Genetic and environmental parameters estimated by REML analysis.

Parameters	Experiment 1		Experiment 2	
	GY	W100S	GY	W100S
$\sigma_{a}^{2}$	96,296.94	57.34	104162.66	18.85
$\sigma_{f}^{2}$	-	-	19279.05	26.17
$\sigma_{b}^{2}$	195,813.99	5.06	6192.58	0.33
$\sigma_{p}^{2}$	360420.77	30.11	108908.36	54.17
$h_{a}^{2}$	0.27	1.90	0.96	0.35
*CV*_*g*_	13.16	5.44	-	-
*CV*_*e*_	40.76	3.86	-	-
*CV*_*r*_	0.33	1.40	-	-

GY–Grain Yield; W100S –Weight of 100 seeds; $\sigma_{a}^{2}$–additive variance; $\sigma_{f}^{2}$–family variance (diallel experiment); $\sigma_{b}^{2}$–variance between plots; $\sigma_{p}^{2}$–phenotypic variance; $h_{a}^{2}$–additive heritability; *CV*_*g*_–coefficient of variation genetic; *CV*_*e*_–coefficient of variation residual; and *CV*_*r*_–ratio between *CV*_*g*_ and *CV*_*e*_.

### Diversity analysis

**Fig 1:**
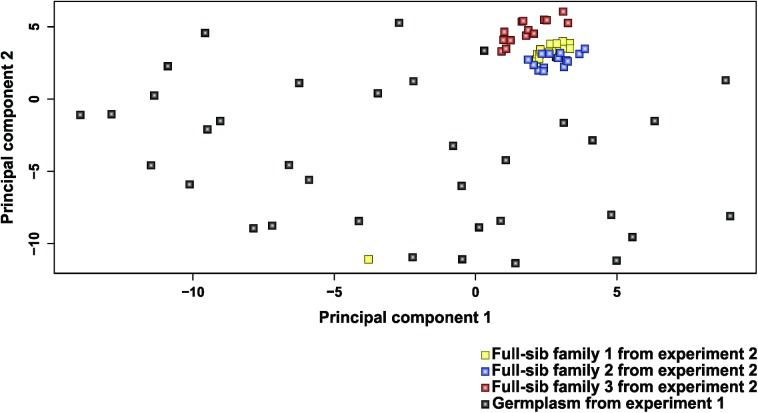
Multidimensional scaling analysis (MDS) showing the first two principal components based on 1,248 markers that were run on the 78 genotypes of Jatropha.

Cluster analysis by MDS showed the diversity between Jatropha genotypes, and only one group was detected (Fig 1). This group was composed of the three full-sib families from the diallel experiment, and all of the genotypes were in this group, except for one from full-sib family 1. In contrast, several genotypes are spread out in the graphic, showing the variability between the genotypes studied.

### Comparison between genomic selection methods

Five-fold cross validation was performed using the full set of 1,248 markers to predict GY and W100S in Jatropha. Prediction ability was estimated as the correlation of GEBV and phenotype values in the validation population.

**Fig 2:**
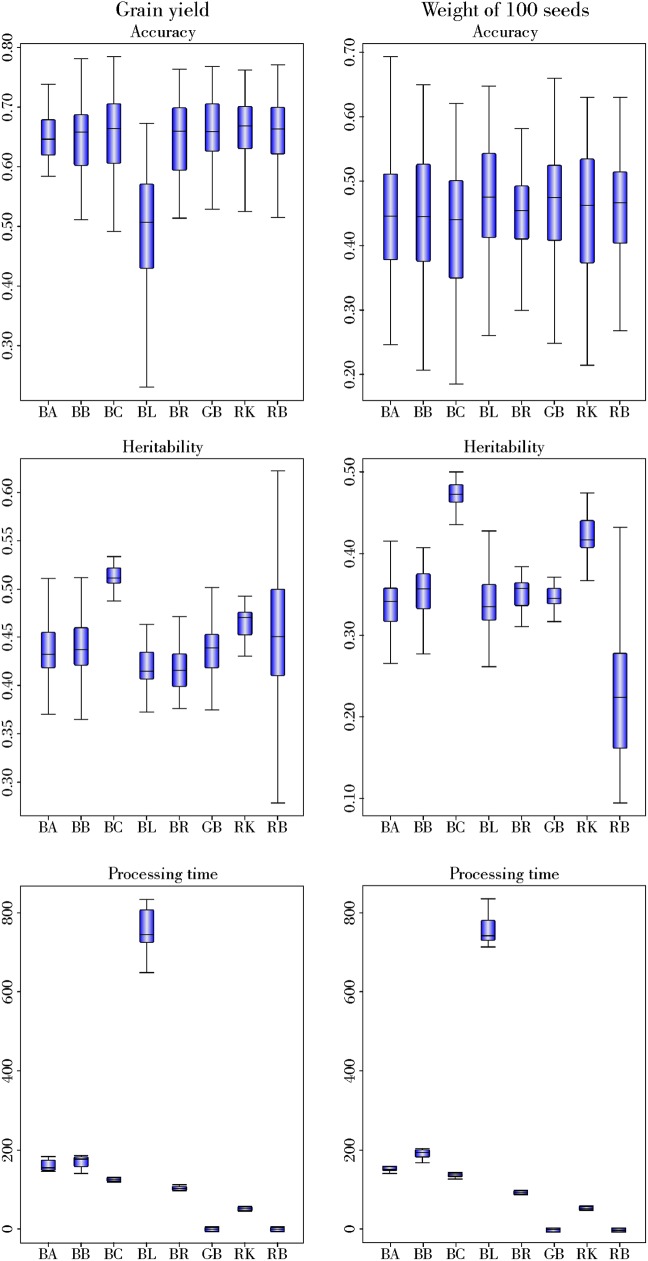
Comparison between genomic selection methods to predict grain yield and weight of 100 seeds. BA- Bayes A; BB–Bayes B; BC–Bayes Cπ; BR–Bayesian Ridge Regression; BL–Bayesian LASSO; GB–G-BLUP; RK–Reproducing kernel Hilbert Space; and RB–RR-BLUP.

The prediction ability was similar between GWS methods for GY and W100S (Fig 2), except BLASSO, which presented smaller values for GY. The average prediction ability of GY (0.66) was higher than W100S (0.46).

There were no differences between the GWS methods to estimate heritability for both traits, except in that Bayes Cπ estimated a higher heritability (Fig 2).

The processing time ranged from 0.08 and 0.07 (RR-BLUP) to 753.09 and 684.23 seconds (BL) for GY and W100S, respectively. We observed that RR-BLUP and G-BLUP were the fastest methods, followed by RKHS: G-BLUP and RR-BLUP were, respectively, 500 and 100 times faster than the fastest Bayesian method (RKHS) and 7000 and 1400 times faster than the slowest Bayesian method (BLASSO).

### Influence of marker density on GWS models prediction

**Fig 3:**
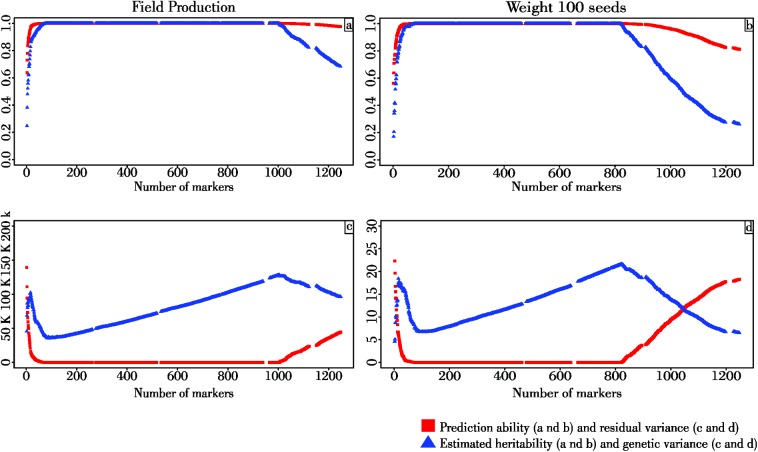
Effect of the number of markers in the prediction ability and estimated heritability (a and b); and genetic and residual variance (c and d) of Grain Yield (GY) and Weight of 100 Seeds (W100S).

The number of markers did not affect the prediction accuracies that presented values close to 1, except when the number of markers was less than 50 or greater than 1,000 (Fig 3A and 3B) for both traits. The estimated heritability showed the same shape; i.e., heritability increased beyond 50 markers, stayed constant until 1,000 and 800 markers for GY and W100S, respectively, and decreased when more than 1,000 and 800 markers were used to train the model (Fig 3A and 3B).

Genetic variance presented a cubic shape, decreasing beyond 100 markers, increasing beyond 1,000 and 800 for GY and W100S, respectively, and decreasing when more than 1,000 and 800 markers were used to train the model (Fig 3C and 3D). The residual variance decreased over 100 markers, coming to values near 0, maintained small values beyond 1,000 and 800 markers for GY and W100S, respectively, and increased thereafter (Fig 3C and 3D).

## Discussion

### Phenotypic analysis

The genetic variance found in our study for GY was similar to those of previous reports [12, 25] while the heritability was greater. The heritability was overestimated in our study because the residual variance was overestimated because the residual variance was calculated as the difference between the phenotypic variance and other components of variance. Then, when the genetic variance is too large and the number of individuals is small, the residual variance can be negative and then the heritability can be greater than 1. This problem can be solved evaluating more plants. However, we could not do it because we had problems with the DNA extraction, and just a few plants could be evaluated. When we estimated these parameters using all plants in the experiment this problem was solved as showed by Junqueira et al. [25] and Peixoto et al. [41].

It was observed that the genetic variance for GY was greater than that for W100S. This difference was possibly due to a scale effect: whereas GY ranged from 0 to 999, W100S ranged from 0 to 96.78.

Another parameter commonly used to evaluate the genetic variability between families is the ratio coefficient of variation (CV_r_) [42]. When the relationship between CV_g_ and CV_e_ is greater than 1, the selection gain will be high [43]. In this study, CV_r_ was greater than 1 for W100S in experiment 1. However, CV_r_ was lower than 1 for GY, which indicated that phenotypic selection may not provide any genetic gain for this trait.

Based on the parameters estimated by REML analysis, selecting superior genotypes for GY and W100S based on phenotypic values would not provide a good selection gain for the next generation because approximately 73% and 65% of the phenotypic variance is not genetic for GY and W100S, respectively. Thus, it is necessary to use more accurate methodologies to predict genetic effects. Therefore, based on these results and on previous research [12, 25], we suggest that the GWS is more appropriate than ANOVA and REML/BLUP to perform analyses and select superior genotypes for Jatropha breeding because the GWS can capture minor genetic differences between families, whereas ANOVA and REML/BLUP cannot.

### Comparison between GWS methods

Several studies have shown that compared with ridge regression methods, more complex statistical methods give only a small increase in the accuracy of genomic prediction for polygenic traits [44–47]. However, this small increase in the prediction accuracy is not sufficient to make Bayesian methods generate statistically better results than G-BLUP. GWS studies conducted in maize, wheat, oat, and barley for both agronomic and disease traits also suggested slight differences among various genomic prediction algorithms [15, 48–50]. In this study, G-BLUP performed similar to all Bayesian methods for GY and W100S. This might be due to the use of non-informative prior distributions in Bayesian methods, resulting in the posterior distribution being influenced solely by the likelihood function. Perhaps meta-analysis can improve accuracies in Bayesian methods by fitting prior distributions using parameters estimated by historical data [51]. Moreover, G-BLUP has other advantages, such as relative simplicity, reduced computing time, and the well-known optimality properties of mixed models for selection [52]. For example, Azevedo et al. [53] analyzed 10 GWS models, including G-BLUP and BLASSO, proposed modifications to the models, and concluded that the G-BLUP, BAYES A*B* (−2,8) and BAYES A*B* (4,6) methods presented the best results and were adequate for accurately predicting genomic breeding. Thus, G-BLUP was chosen to perform other analyses.

Moreover, it has now been demonstrated that predictive models built on the basis of genome-wide markers allow breeders to obtain higher selective accuracy, even for traits of low heritability, such as GY and seed oil content in Jatropha. In addition, genomic breeding values may be estimated at the seedling stage, which can reduce the Jatropha breeding cycle by at least five years (6 years for breeding cycles with GWS versus 12 years for breeding cycles without GWS). Because selection response is inversely proportional to breeding cycle length, we calculated the expected impact of GWS on Jatropha breeding. Considering the accuracies and cycles reported here, GWS may increase the selection efficiency in Jatropha breeding by more than 100% [20, 24, 54]. Technow et al. [55], to show how GWS can override phenotypic selection, proposed a formula to estimate the response to indirect selection, being: LY<(rAHX)Lx; where L_Y_ is the cycle length of GWS, r_A_ is the genomic prediction accuracy, H_X_ is the phenotypic selection accuracy, and L_X_ is the cycle length of phenotypic selection. Substituting the values estimated for GY and W100S in the formula, it can be observed that GWS must be superior to phenotypic selection if the cycle length of GWS is the same cycle length of phenotypic selection for GY and less than 49% the cycle length of phenotypic selection for W100S. Therefore, because the cycle is half the length when using GWS (6 years = 1 year for crossing and 5 years to evaluate in different environments) instead of a traditional breeding cycle (12 years = 7 years for crossing and 5 years to evaluate in different environments), GWS is a useful tool to reduce the breeding cycle.

In the case of perennial crops such as Jatropha, they need several years, ranging from 10 to 14 years, to obtain suitable phenotypic evaluations. Based on practical considerations and the theoretical equation presented above, GWS may improve the efficiency of breeding programs. The main step in which GWS will be useful is shortening the length of the breeding cycle. This will occur because the progeny testing phase can be omitted when GWS is applied, and breeders will thus be able to perform early selection at the seedling stage. Then, selected individuals can be immediately propagated by micropropagation protocols; consequently, optimized clonal trials with several years of anticipation can be established compared to a classical breeding.

The selection response per time unit may be drastically increased (by as much as 50%) when the breeding cycle is reduced because the selection response is inversely proportional to the breeding cycle length, as theoretically and experimentally demonstrated [20]. For instance, simulation studies for oil palm have demonstrated that GWS can be more effective than phenotype selection in terms of both cost and time reduction because breeders can perform four breeding cycles in the same period of time when using GWS instead of the two breeding cycles that are permitted when traditional breeding is used [19, 24]. Moreover, with the development of genotyping-by-sequencing approaches, early selection may also allow breeders to increase selection intensity, thus allowing them to have a large number of individuals quickly genotyped for thousands of markers at a low cost. Additionally, experiments in forest breeding are usually limited in size due to economic and operational aspects, which reduces both the number of evaluated individuals and the accuracy of phenotypic selection. Therefore, breeders will be able to reduce their investment in field-testing using GWS by evaluating just a few individuals that will be used to train the model, thereby saving time and resources and improving the selection precision for traits of low heritability.

### Influence of marker density on GWS models prediction

The effectiveness of GWS depends on the correlation between the predicted genotypic value and the underlying true genotypic value [56]. This correlation, also called the prediction ability, of GWS has been expressed as a function of the marker density, training population size (N), trait heritability on an entry-mean basis (h^2^), and the effective number of quantitative trait loci (QTL) or effective number of chromosome segments underlying the trait (Me) [57, 58]. Simulation and cross-validation studies have indicated that prediction accuracy generally increases as h^2^ increases [18, 23, 49] and is not affected when the number of markers increases [59, 60]. Peixoto et al. [12] showed, using REML/BLUP, that the most important traits in Jatropha have different heritabilities, such as GY, oil content, phorbol ester concentration, and W100S, the heritabilities of which were 0.32, 0.24, 0.71 and 0.85, respectively. Therefore, different strategies should be developed to use GWS in Jatropha and obtained high prediction abilities for those traits.

Models fitted using over 1000 and 800 markers were capable of predicting GY and W100S, respectively (Fig 3). The comparable performance of a limited number of markers (1000 and 800) relative to the complete marker data set could be due to marker saturation because random markers with uniform coverage across each chromosome were selected. With a larger linkage disequilibrium, the addition of more markers will not increase the accuracy of the predictive models [61]. Because a linear correlation between the number of markers and prediction accuracy was not observed in this research, a good GWS model for predicting GY and W100S in Jatropha can be fitted by using approximately 1000 and 800 markers, respectively, in a diverse genotype collection. The use of a small SNP set can lead to cost savings. Using a uniform or common SNP set will allow the consistent use of genome-wide prediction in research and breeding programs.

However, before Jatropha breeding programs incorporate GWS on a large scale, the results found in our study must be validated across years and by evaluating progenies. Because the genetic material used in this study consisted of diverse accessions from the germplasm bank (Fig 1), the results from the genetic structure and composition of entries in this study would be applicable to germplasm enhancement programs using diverse collections to obtain parental materials.

### Future GWS applications in Jatropha

Because the demand for biodiesel is constantly increasing, the development of dedicated crops has been suggested as a strategic action. Thus, biodiesel production is expected to become much more efficient if not only conversion processes themselves are improved but also oil feedstocks are optimized to this end. In that context, genomics offers innumerable technologies for collecting genetic information that could be potentially integrated into Jatropha breeding to aid in the development of cultivars with outstanding performance for biodiesel production. Because genomics offers a platform to learn more about the relationships of genes and phenotypes, the long-term goal of applying genomics to breeding is to link genomic information with the field research that is currently underway, with the purpose of developing accurate predictive models. Such models could then be operationally used by breeders to estimate the performance and adaptability of genotypes across locations or ecosystems based on genetic data alone, i.e., without the need for conducting laborious and expensive phenotyping trials at the beginning of the breeding cycle. In the context of a long-lived perennial crop, with long breeding cycles and late-expressing traits, the achievement of such a long-term goal promises to revolutionize selective breeding [62]. Because some of the most promising feedstocks for biodiesel production, such as Jatropha, oil palm, macaw palm (*Acrocomia aculeate*), and pongamia (*Pongamia pinnata*), are perennial crops, genomic breeding is one of the most promising ways to foster the development of perennial crops dedicated to biodiesel production.

In the near future, GWS can improve the efficiency of producing Jatropha oil, but many studies are needed to prove all such theories in practice experiments because no study to date has evaluated GWS in biofuel traits. Therefore, studies should evaluate how the GWS method is better to capture high accuracy for oil production, how many individuals and markers are needed to train the model, and how the GxE interaction can influence prediction accuracy.

## Conclusion

There was genetic variance between the genotypes evaluated, and it was possible to obtain selection gain using GWS;

All genomic selection methods tested in this study can be used to predict the grain yield and weight of 100 seeds in Jatropha;

Training models fitted using 1,000 and 800 markers are sufficient to capture the maximum genetic variance and, consequently, the maximum prediction ability for grain yield and weight of 100 seeds, respectively.

## Supporting information

S1 Table Identification and origin for each accession used in the germplasm bank experiment. (DOCX)

S2 Table Identification of families used in the diallel experiment. (DOCX)

S3 Table Data set used to perform all genomic wide selection analyses in this study. (DOCX)
