# Supplementary material for: Breeding Jatropha curcas by genomic selection: A pilot assessment of the accuracy of predictive models
Source: PLoS One. 2017 Mar 15;12(3):e0173368. doi: 10.1371/journal.pone.0173368 (PMC5351973; doi:10.1371/journal.pone.0173368)
Supplement: S1 Table — (DOCX) [file pone.0173368.s001.docx]

**S1 Table**. Identification and origin for each accession used in germoplasm bank experiment.

| **Accession** | **Origin** |
| --- | --- |
| 101 | Rio Verde-GO |
| 102 | Santa Vitoria-MG |
| 103 | Santa Vitoria-MG |
| 104 | Santa Vitoria-MG |
| 105 | Santa Vitoria-MG |
| 106 | Santa Vitoria-MG |
| 107 | Santa Vitoria-MG |
| 108 | Santa Vitoria-MG |
| 110 | Santa Vitoria-MG |
| 111 | Santa Vitoria-MG |
| 112 | Petrolina-PE |
| 113 | Sete Lagoas-MG |
| 114 | Umuarama-PR |
| 115 | Xambrê-PR |
| 116 | S. Jose dos Campos-SP |
| 117 | S. Franc. do Gloria-MG |
| 118 | Curvelo-MG |
| 120 | Guapimirim-RJ |
| 121 | Bom Jesus-RJ |
| 122 | Belmiro Braga-MG |
| 123 | Belmiro Braga-MG |
| 124 | Maranhão |
| 125 | Maranhão |
| 126 | Maranhão |
| 127 | Maranhão |
| 128 | Maranhão |
| 129 | Maranhão |
| 130 | Maranhão |
| 131 | Maranhão |
| 132 | Maranhão |
| 133 | Maranhão |
| 134 | Maranhão |
| 136 | Maranhão |
| 137 | Maranhão |
| 138 | Maranhão |
| 139 | Maranhão |
| 140 | Maranhão |
| 141 | Maranhão |
| 142 | Maranhão |
| 143 | Maranhão |
| 145 | Maranhão |
| 147 | Maranhão |
| 148 | Candeias-BA |
| 149 | Paraná |
| 150 | Janaúba-MG |
| 151 | Janaúba-MG |
| 152 | Janaúba-MG |
| 153 | Janaúba-MG |
| 154 | Janaúba-MG |
| 155 | Nova Porteirinha-MG |
| 156 | Nova Porteirinha-MG |
| 157 | Nova Porteirinha-MG |
| 158 | Nova Porteirinha-MG |
| 159 | Nova Porteirinha-MG |
| 160 | Nova Porteirinha-MG |
| 161 | Nova Porteirinha-MG |
| 163 | Nova Porteirinha-MG |
| 164 | Nova Porteirinha-MG |
| 165 | Nova Porteirinha-MG |
| 166 | Nova Porteirinha-MG |
| 167 | Nova Porteirinha-MG |
| 168 | Janaúba-MG |
| 169 | Jaiba-MG |
| 170 | Jaiba-MG |
| 172 | João Pinheiro-MG |
| 174 | João Pinheiro-MG |
| 175 | João Pinheiro-MG |
| 176 | João Pinheiro-MG |
| 177 | João Pinheiro-MG |
| 178 | João Pinheiro-MG |
| 179 | João Pinheiro-MG |
| 180 | João Pinheiro-MG |
| 181 | João Pinheiro-MG |
| 182 | João Pinheiro-MG |
| 183 | Jaiba-MG |
| 185 | Pelotas-RS |
| 186 | Pelotas-RS |
| 187 | Pelotas-RS |
| 188 | Curvelo-MG |
| 189 | Sete Lagoas-MG |
| 190 | Curvelo-MG |
| 191 | S. Franc. de Assis-RS |
| 192 | Lavras-MG |
| 193 | Lavras-MG |
| 194 | Lavras-MG |
| 195 | Lavras-MG |
| 196 | Lavras-MG |
| 198 | Lavras-MG |
| 199 | Lavras-MG |
| 200 | Lavras-MG |
| 201 | Lavras-MG |
| 202 | Lavras-MG |
| 203 | Lavras-MG |
| 204 | Lavras-MG |
| 205 | Lavras-MG |
| 206 | Lavras-MG |
| 207 | Lavras-MG |
| 208 | Lavras-MG |
| 209 | Lavras-MG |
| 210 | Lavras-MG |
| 211 | Lavras-MG |
| 212 | Lavras-MG |
| 214 | Ribeirão Preto-SP |
| 215 | S. Franc. do Gloria-MG |
| 216 | S. Miguel do Araguaia-GO |
| 217 | S. Miguel do Araguaia-GO |
| 218 | S. Miguel do Araguaia-GO |
| 219 | S. Miguel do Araguaia-GO |
| 220 | Paraná |
| 221 | Paraná |
| 222 | Paraná |
| 223 | Paraná |
| 224 | S. Franc. do Glória-MG |
| 225 | Agua de Santa Barb.-SP |
| 226 | Agua de Santa Barb.-SP |
| 227 | Agua de Santa Barb.-SP |
| 228 | Barra dos Bugres-MT |
| 229 | Barra dos Bugres-MT |
| 230 | Petrolina-PE |
| 231 | Apucarana-PR |
| 232 | Apucarana-PR |
| 233 | Pirassununga-SP |
| 234 | Arinos-MG |
| 235 | Arinos-MG |
| 236 | Minas Gerais |
| 237 | Minas Gerais |
| 238 | Minas Gerais |
| 239 | Minas Gerais |
| 240 | Minas Gerais |
| 241 | Minas Gerais |
| 243 | Minas Gerais |
| 244 | Arinos-MG |
| 247 | Minas Gerais |
| 250 | Minas Gerais |
| 251 | Minas Gerais |
| 252 | Minas Gerais |
| 253 | Minas Gerais |
| 254 | Minas Gerais |
| 255 | Minas Gerais |
| 256 | Uberlandia-MG |
| 257 | Uberlandia-MG |
| 258 | Uberlandia-MG |
| 260 | Jales-SP |
| 261 | Jales-SP |
| 262 | Porangatu-GO |
| 263 | Porangatu-GO |
| 264 | Dourados-MS/CPAO |
| 265 | Dourados-MS/CPAO |
| 266 | Minas Gerais |
| 267 | Minas Gerais |
| 268 | Minas Gerais |
| 269 | Minas Gerais |
| 270 | Minas Gerais |
| 271 | Minas Gerais |
| 272 | Minas Gerais |
| 274 | Minas Gerais |
| 275 | Minas Gerais |
| 276 | Minas Gerais |
| 277 | Minas Gerais |
| 278 | Minas Gerais |
| 279 | Minas Gerais |
| 280 | Minas Gerais |
| 281 | Minas Gerais |
| 282 | Minas Gerais |
| 283 | Minas Gerais |
| 289 | Minas Gerais |
| 290 | Minas Gerais |
| 297 | Sertanópolis-PR |
| 298 | Sidrolandia-MS |
| 299 | Rio Grande do Sul |
| 300 | Rio Grande do Sul |
| 302 | Rio Grande do Sul |
| 303 | Campina Grande-PB |
| 304 | Campina Grande-PB |
| 309 | Pará |
| 310 | Pará |
| 312 | Unknown |
| 313 | Unknown |
| 315 | Unknown |

GO – Goiás; MG – Minas Gerais, PB - Paraíba; SP – São Paulo; PR – Paraná; MS – Mato Grosso do Sul; PE – Pernambuco; MT – Mato Grosso; RS – Rio Grande do Sul; BA – Bahia; RJ – Rio de Janeiro. All of those are Brazilian States were those accessions were collected.
