# Supplementary material for: Breeding Jatropha curcas by genomic selection: A pilot assessment of the accuracy of predictive models
Source: PLoS One. 2017 Mar 15;12(3):e0173368. doi: 10.1371/journal.pone.0173368 (PMC5351973; doi:10.1371/journal.pone.0173368)
Supplement: S2 Table — (DOCX) [file pone.0173368.s002.docx]

**S2 table.** Identification of families used in the diallel experiment.

| **Female Parent** | **Male Parent** | **Crossing identification** |
| --- | --- | --- |
| Nontoxic & susceptible | Toxic & susceptible | 6 |
| Nontoxic & susceptible | Toxic & resistant | 7 |
| Toxic & susceptible | Toxic & resistant | 10 |
