# Supplementary material for: Breeding Jatropha curcas by genomic selection: A pilot assessment of the accuracy of predictive models
Source: PLoS One. 2017 Mar 15;12(3):e0173368. doi: 10.1371/journal.pone.0173368 (PMC5351973; doi:10.1371/journal.pone.0173368)
Supplement: S3 Table — (DOCX) [file pone.0173368.s003.docx]

**S3 table**. Data set used to perform all genomic wide selection analyses in this study.

| **Identification** | **W100SS** | **PROD** |
| --- | --- | --- |
| 101* | 80.8 | 1187 |
| 107 | 68.9 | 1003 |
| 111 | 74.9 | 620 |
| 121 | 74.8 | 1002 |
| 123 | 68.7 | 526 |
| 132 | 71.3 | 1372 |
| 134 | 64.3 | 779 |
| 141 | 71.5 | 723 |
| 143 | 74.4 | 802 |
| 157 | 67.5 | 1722 |
| 160 | 70.3 | 1105 |
| 168 | 68.8 | 2041 |
| 169 | 76.5 | 407 |
| 170 | 74.8 | 148 |
| 171 | 61.8 | 1112 |
| 183 | 70.7 | 251 |
| 185 | 66.6 | 1131 |
| 190 | 63.7 | 1697 |
| 192 | 68.7 | 1689 |
| 198 | 72 | 978 |
| 215 | 69.3 | 1420 |
| 219 | 68.8 | 1052 |
| 221 | 72.1 | 753 |
| 222 | 73.1 | 1068 |
| 224 | 74.1 | 694 |
| 228 | 72.4 | 369 |
| 240 | 68.1 | 271 |
| 252 | 66.2 | 959 |
| 259 | 77.2 | 531 |
| 265 | 72.9 | 609 |
| 269 | 62.9 | 644 |
| 270 | 61.1 | 1015 |
| 273 | 65.4 | 809 |
| 276 | 62.1 | 676 |
| 289 | 70.4 | 1289 |
| 290 | 67.8 | 911 |
| 10_1 | 74.6 | 477 |
| 10_2 | 78.7 | 434 |
| 10_3 | 71.7 | 427 |
| 10_4 | 79.1 | 644 |
| 10_5 | 78.7 | 1160 |
| 10_6 | 71.4 | 702 |
| 10_7 | 74.3 | 550 |
| 10_8 | 66.6 | 465 |
| 10_9 | 72.4 | 448 |
| 10_10 | 69.9 | 975 |
| 10_11 | 82.1 | 665 |
| 10_12 | 69.1 | 389 |
| 10_13 | 73.8 | 335 |
| 10_14 | 74 | 218 |
| 6_1 | 78.5 | 1473 |
| 6_2 | 81.4 | 1504 |
| 6_3 | 80.8 | 1297 |
| 6_4 | 70.33 | 1658 |
| 6_5 | 74.3 | 1408 |
| 6_6 | 76 | 1770 |
| 6_7 | 65.4 | 1190 |
| 6_8 | 66 | 1767 |
| 6_9 | 70.7 | 1451 |
| 6_10 | 70 | 627 |
| 6_11 | 64.9 | 1139 |
| 6_12 | 71.1 | 1788 |
| 6_13 | 76.7 | 1548 |
| 6_14 | 77.5 | 1462 |
| 7_1 | 74.17 | 524 |
| 7_2 | 77 | 525 |
| 7_3 | 77.4 | 436 |
| 7_4 | 77.4 | 667 |
| 7_5 | 81.3 | 408 |
| 7_6 | 68.9 | 284 |
| 7_7 | 74.7 | 687 |
| 7_8 | 77.46 | 345 |
| 7_9 | 75 | 168 |
| 7_10 | 88.3 | 176 |
| 7_11 | 87.2 | 422 |
| 7_12 | 82.09 | 288 |
| 7_13 | 67 | 1036 |
| 7_14 | 71.6 | 422 |

*101 to 290: Half-sib families chosen from germplasm bank experiment. 6, 7 and 10: Full-sib families from diallel experiment. Numbers 1 to 14 are plants evaluated within each full-sib family.
